# SnoRNA and lncSNHG: Advances of nucleolar small RNA host gene transcripts in anti-tumor immunity

**DOI:** 10.3389/fimmu.2023.1143980

**Published:** 2023-03-15

**Authors:** Hao Xiao, Xin Feng, Mengjun Liu, Hanwen Gong, Xiao Zhou

**Affiliations:** ^1^ Department of Clinical Laboratory Medicine, The Third Xiangya Hospital, Central South University, Changsha, Hunan, China; ^2^ Department of Clinical Laboratory Medicine, Xiangya School of Medicine, Central South University, Changsha, Hunan, China

**Keywords:** snoRNA, lncSNHG, anti-tumor immunity, immune cell, nucleolar small RNA host gene

## Abstract

The small nucleolar RNA host genes (SNHGs) are a group of genes that can be transcript into long non-coding RNA SNHG (lncSNHG) and further processed into small nucleolar RNAs (snoRNAs). Although lncSNHGs and snoRNAs are well established to play pivotal roles in tumorigenesis, how lncSNHGs and snoRNAs regulate the immune cell behavior and function to mediate anti-tumor immunity remains further illustrated. Certain immune cell types carry out distinct roles to participate in each step of tumorigenesis. It is particularly important to understand how lncSNHGs and snoRNAs regulate the immune cell function to manipulate anti-tumor immunity. Here, we discuss the expression, mechanism of action, and potential clinical relevance of lncSNHGs and snoRNAs in regulating different types of immune cells that are closely related to anti-tumor immunity. By uncovering the changes and roles of lncSNHGs and snoRNAs in different immune cells, we aim to provide a better understanding of how the transcripts of SNHGs participate in tumorigenesis from an immune perspective.

## Introduction

Non-coding RNAs (ncRNAs) regulate gene expression at the transcriptional, RNA processing, and translation levels (1). Small nucleolar RNAs (snoRNAs) are a class of ncRNAs ranging from 60 to 300 nt in length and are closely associated with the splicing and processing of ribosomal RNA (rRNA) precursors, post-transcriptional modification processes, and ribosome biosynthesis (2). Most snoRNAs are encoded in introns of protein-coding and non-protein-coding genes, called Small Nucleolar RNA Host Genes (SNHGs). The primary SNHGs RNA transcripts, which include all exons and introns and their snoRNAs, are cleaved into different parts: introns are processed into snoRNAs and mainly function in the nucleolus, exons are re-spliced and function in the cytoplasm, and the full-length transcript, including exons, is retained and functions as Long Non-Coding RNA SNHGs (lncSNHGs) (3–6).

The dysregulation of snoRNAs and lncSNHGs in a variety of cancers has attracted increasing interest, as they affect tumor development through multiple mechanisms and can be linked to clinic pathology. From a tumorigenesis perspective, the risk of developing tumors increases due to the mutations in snoRNAs and upregulation of lncSNHGs expression (7–9). From the perspective of tumor development, snoRNAs and lncSNHGs are involved in regulating the malignant biological behavior of tumor cells such as Epithelial-Mesenchymal Transition (EMT), cell cycle progression, proliferation, invasion, and evasion of apoptosis (10–14). From a clinicopathological point of view, snoRNA and its derived fragments as well as lncSNHG are associated with clinical outcomes in patients (15–17).

The immune system is also closely involved in tumor development. Studies have shown that snoRNAs and their host genes play a crucial role in anti-tumor immunity. As the understanding of the involvement of snoRNAs and their host genes in the immune regulation of tumors continues to improve, the use of snoRNAs and lncSNHGs in immunodiagnosis, especially in the prognosis of clinical patients, and in immunotherapy is becoming widespread (18–21).

In this paper, we review the expression and key roles of snoRNA and lncSNHG in distinct immune cell types. By summarizing the up-to-date research on snoRNA and lncSNHG in immune cells and immune modulation, we further discuss the roles of these two ncRNAs in anti-tumor immunity. In addition, we have further reviewed the clinical implications of snoRNAs and lncSNHGs on patient prognosis and discussed the potential clinical applications of snoRNA and lncSNHG as therapeutic and diagnostic targets in patients with cancer.

## SNHG transcripts: snoRNA and lncSNHG

**Figure 1:**
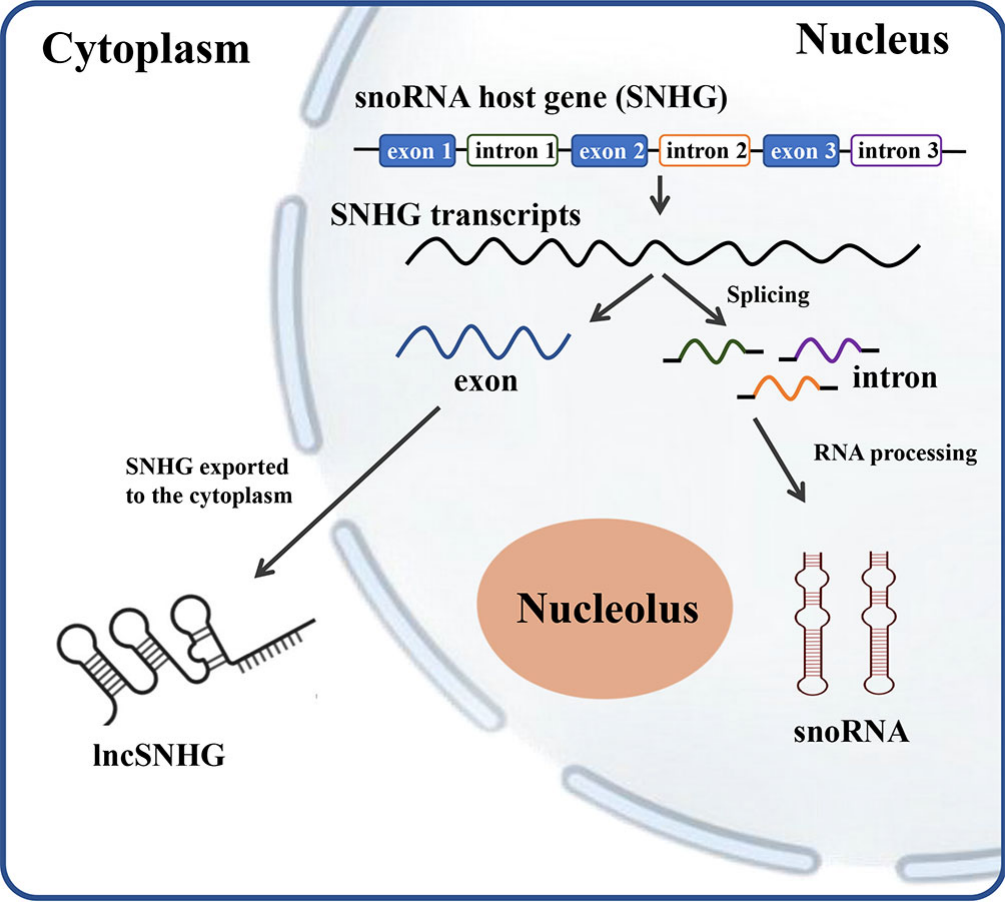
The scheme of relationship between SNHG, snoRNA and lncSNHG. The majority of small nucleolar RNA host genes (SNHGs) are protein-coding and non-protein-coding Genes. SNHGs are transcript into the primary SNHG transcripts at nucleus. The primary SNHG transcripts containing all exons and introns with their snoRNAs are cut into different exons and introns. Exons are then re-spliced and translocated into cytoplasm to function as protein coding mRNA or non-coding RNA (lncSHNG). Intronic sequences are further processed into mature snoRNA and then assembled into small nucleolar ribonucleoprotein particles (snoRNPs), which is followed by localization to Cajal bodies and nucleolus. Exon, mRNA or lncRNA exons; intron, mRNA introns that contains the intronic snoRNA.

SNHG encodes for the production of full-length transcripts, including exons and introns, which play a vital role in tumor proliferation, migration, and infiltration (5). The transcripts containing all exons and introns of SNHGs without coding potentials are referred to as lncSNHG, which is transported into cytoplasm and regulates multiple bioprocesses. The nuclear transcripts of SNHG, mostly the introns, were further processed into smaller molecules with a length between 65 to 300 nt, which is defined as snoRNA (**Figure 1**).

LncSNHG is an emerging class of regulators of gene transcription that plays key roles in the development and progression of cancer, acting as oncogenes or tumor inhibitors (22, 23). It is reported that lncSNHGs are overexpressed in human tumors and can induce proliferation, invasion, and metastasis (24). SNHG1, SNHG3, SNHG4, SNHG6, SNHG7, SNHG12, SNHG14, SNHG16, SNHG17, SNHG20 and SNHG22 act as oncogenes to promote tumor growth, while SNHG9 acts as a tumor suppressor gene. In addition, SNHG5 and SNHG15 play a dual role (6).

**Figure 2:**
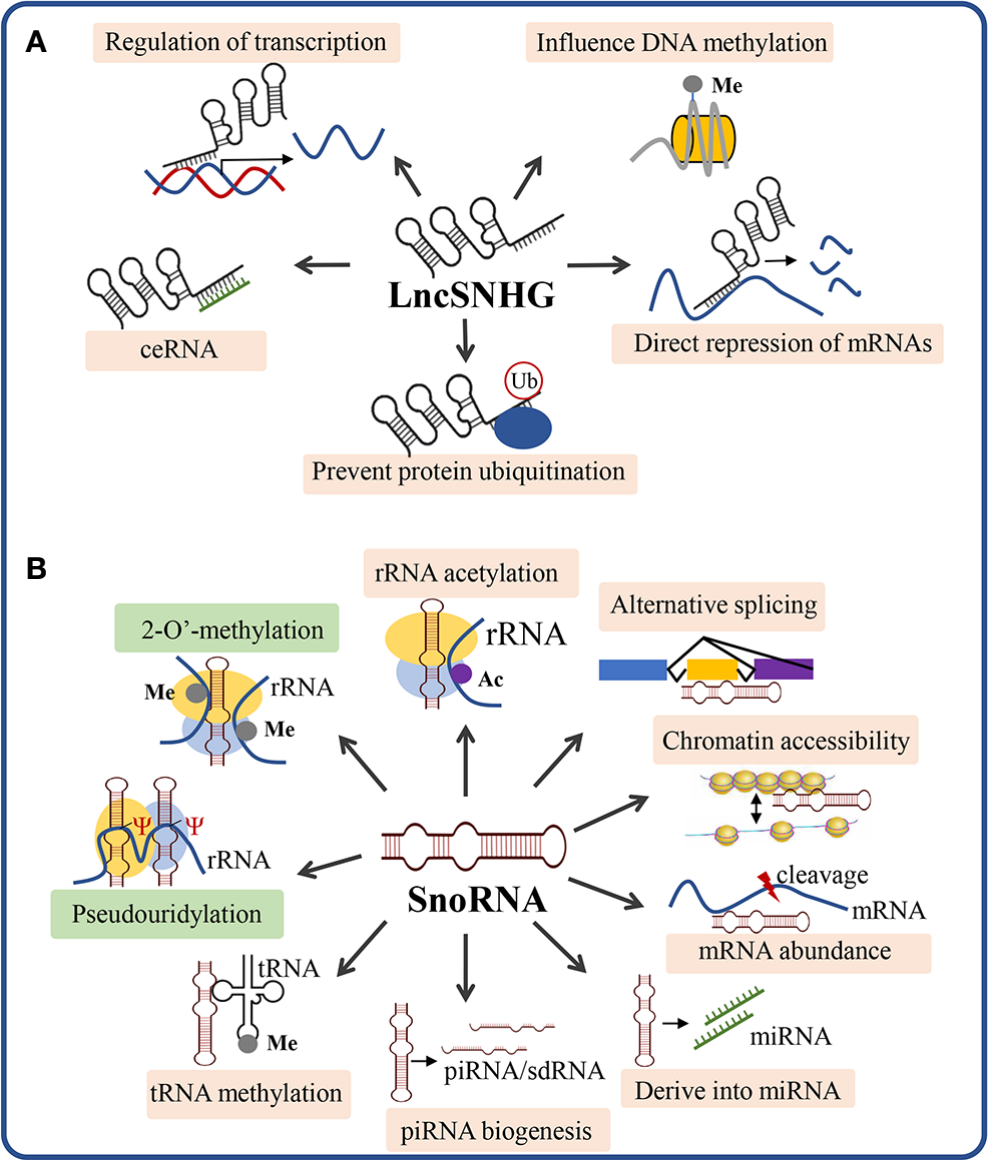
Scheme of the mechanism of action of lncSNHG and snoRNA. **(A)** The 5 most studied mechanism of lncSNHGs to exert their regulatory functions. Ub, ubiquitination; Me, methyl. **(B)** The canonical and non-canonical mechanisms underlying snoRNA-mediated bioactivities. Green boxes show the canonical mechanisms of snoRNA to regulate the biosynthesis and modification of rRNA, tRNA, and snRNA. The orange boxes show the non-canonical mechanisms of snoRNA. Ac, acetyl; Me, methyl; Ψ, pseudouridylic acid.

LncSNHGs participate in cancer regulation mainly through five molecular mechanisms of action (**Figure 2A**): 1) affecting DNA methylation, 2) interacting with transcription factors, 3) acting as competing endogenous RNAs (ceRNAs), 4) directly binding to mRNAs and inhibiting translation, 5) interacting with proteins to prevent protein ubiquitination (25). The mechanisms of lncSNHGs rely on their cellular localization, while the nuclear localization of lncSNHGs mainly influences DNA methylation and transcription, and the cytoplasm localization mainly influences the bioactivity of mRNA and protein (25).

SnoRNAs are predominantly released from the intronic sequences of SNHGs by splicing, while a minority of snoRNAs are derived from intergenic regions (26). A growing number of research reported that snoRNA originate from the introns of ribosomal protein-coding genes such as the C/D box snoRNA family arising from RPL10(U70) (27), RP113A(U32 toU35), RPL17A(U36), RPS8(U38 to U40) (28), and from other protein-coding genes such as EEF2(U37) (28), NSD2(ACA11) (29). To date, there are 232 SNHGs found in the human genome that host the snoRNAs, among which 15 of these host genes are non-protein coding (30). It was reported that a specific lncSNHG is usually positively correlated with the corresponding snoRNA (31), the correlation between lncSNHGs and snoRNA is summarized in **Table 1**.

**Table 1:** SnoRNA in relation to SNHG.

SNHG	snoRNA
**SNHG1**	SNORD22 (32) SNORD25, SNORD26, SNORD27, SNORD28, SNORD29, SNORD30, SNORD31 (3)
**SNHG2(GAS5)**	U44, U47, U74, U75, U76, U77, U78, U79, U80, U81 (33)
**SNHG3**	SNORD17 (34)
**SNHG4**	SNORA74A, SNORA74 (35)
**SNHG5**	SNORD 50, SNORD50′ (36)
**SNHG6**	U87 SNORD (37), U88 small Cajal bodies (38)
**SNHG7**	SNORA17 and SNORA43 (39)
**SNHG8**	SNORA24 (40)
**SNHG11**	SNORA71E, SNORA39 (41)
**SNHG12**	SNORA44, SNORA61, SNORA16A, and SNORD99 (42)
**SNHG14**	SNORD116 (43)
**SNHG15**	SNORA 9 (44)
**SNHG16**	snoRD1A, snoRD1B, and snoRD1C (45)
**SNHG17**	SNORA71A (46)
**SNHG20**	SCARNA16 (47)

According to the presence of conserved sequences(“box”), snoRNAs are generally classified into three classes, namely C/D box snoRNA (SNORD), H/ACA box snoRNA (SNORA), and scaRNA (SCANRA) (48). SNORDs contain a box C (motif RUGAUGA) and a box D (motif CDGA) and a less conserved box C’ and box D’ (49). The SNORDs form a closed loop and bind to fibrillarin, NOP56 (NOL5A), NOP5/NOP58, and NHP2L1 to form small nucleolar ribonucleoparticles (snoRNPs) (20, 27, 50). The majority of SNORDs carry specific sequences which are complementary to other RNAs and thus can guide 2-O’-methylation (box C/D) to the targeted RNA (51). The SNORAs contain the H box (motif ANANNA, N represents any NT) and the ACA box (trinucleotide ACA). The box H/ACA snoRNAs form two stem loops and bind with dyskerin, GAR1 (NOLA1), NHP2 (NOLA2), and NOP10 (NOLA3) to form stable snoRNPs (50). SNORAs guide pseudouridylation (box H/ACA) to the targeted RNAs by the sequences that are complementary to other RNAs thus regulating rRNA and small nuclear RNAs (snRNA) modification (51). SNORDs with a long UG repeat and SNORAs with an additional CAB box(motif UGAG) are classified into the third class of snoRNAs, which is the Cajal body–specific RNAs (scaRNAs) and involved in the 2’-O-ribose methylation and pseudouridylation of small nuclear RNAs of the spliceosome (52, 53). In addition, there is also a subclass of C/D box snoRNA that has no target RNA and does not recognize specific sequences in a base-paired manner, which is known as orphan snoRNA and plays an important role in a variety of physiological and pathological processes (54, 55). Although these orphan snoRNAs seem not to match any other RNAs, they can still function to carry out regulatory functions *via* pre-mRNA splicing, regulation of polyadenylation site (PAS) recognition, disturbing ribosome and snRNA formation, serving as chromatin-associated RNAs, and processing to miRNAs or Piwi-interacting RNAs (piRNAs) (49, 56).

SnoRNAs exert their function *via* canonical and non-canonical mechanisms (**Figure 2B**). The canonical mechanism of snoRNA is mainly involved to guide the chemical modification of ribosomal RNAs, transfer RNAs, and small nuclear RNAs, which play critical roles in ribosome biogenesis and post-transcriptional modifications of RNAs (57). Beyond the canonical functions, snoRNAs can also function *via* non-canonical mechanisms, which include: 1) snoRNA-guided rRNA acetylation, 2) snoRNA-guided tRNA methylation, 3) regulation of piRNA biogenesis, 4) regulation of mRNA abundance, 5) snoRNA-derived miRNA, 6) regulation of alternative splicing, 7) chromatin compaction and accessibility (57). In the following section, we will discuss how snoRNA exerts its functions to regulate the immune cell function, thus influencing anti-tumor immunity.

## snoRNA-mediated immune cell function to participate in tumor immunity

**Figure 3:**
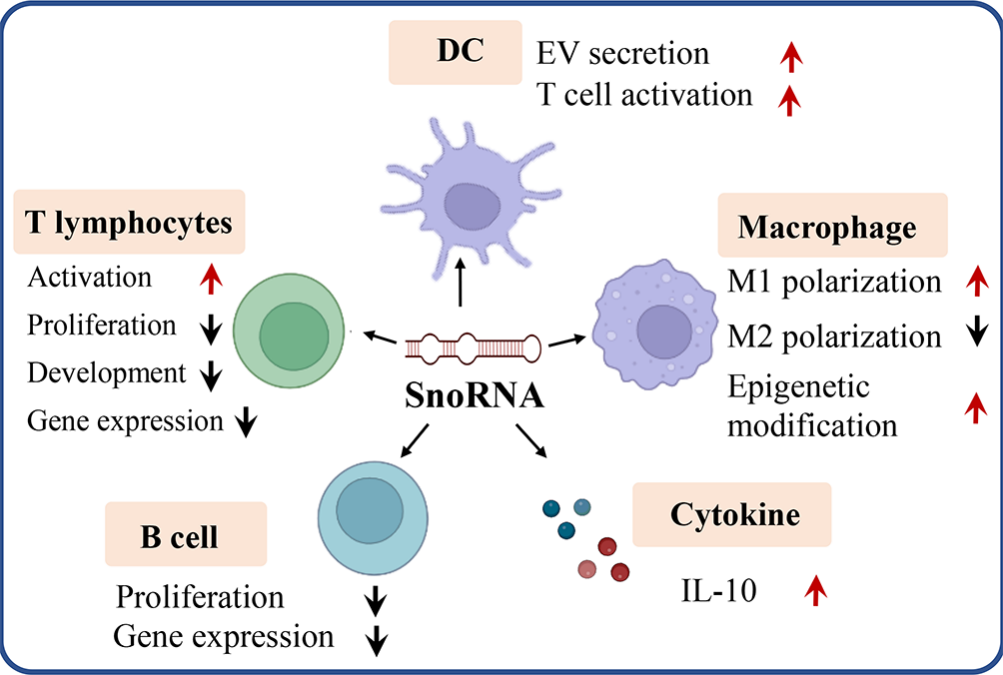
SnoRNA regulates varieties of immune cells (e.g. T lymphocytes, B lymphocytes, dendritic cell, macrophage) as well as cytokine like IL-10 to influence tumorigenesis and tumor immunity. Red arrow, upregulation, black arrow, down regulation.

As a pivotal non-coding RNA type, snoRNA was found to play key roles in tumorigenesis and tumor immunity (57, 58). Dysregulation of snoRNA expression was found in cancer patients to exacerbate tumorigenesis. The comprehensive functions of snoRNAs were reviewed elsewhere (59). It is well established that snoRNAs can be oncogenic and tumor suppressive, the function relies on their role in different oncogenic pathways. SNORD126 is reported to be up-regulated and activates the PI3K-AKT pathway to promote tumor growth (60), and SNORD50A and SNORD50B function as tumor suppressors *via* repressing the activity of K-Ras pathway (61). SnoRNAs are also found to bind with the rRNA complex to regulate cancer-related gene expression. SNORA18L5 was reported to change the localization of RPL5 and RPL11 to regulate p53-dependent tumorigenesis (62). It was reported that substantial downregulation or upregulation of snoRNAs in meningiomas, and the differentially expressed snoRNA are involved in the control of cell survival by inducing or sensitizing cells to apoptosis (63–65). As key players in tumorigenesis and tumor microenvironment (TME), snoRNAs also mediate the functions of many types of immune cells. Here, we discuss the roles of snoRNAs in regulating the behaviors and functions of different immune cells (**Figure 3**).

### T cell

SnoRNA has been found to play an important role in T lymphocyte activation, proliferation, development, and gene expression. Activation of the T cell receptor (TCR) causes massive mRNA expression and ribosome biogenesis that drives metabolic reprogramming, rapid proliferation and differentiation to generate effector populations. Galloway et al. reported that TCR activation results in the m^7^G cap methyltransferase, RNMT, upregulation to induce snoRNA and rRNA production to regulate ribosome biogenesis and gene translation, thereby regulating CD4^+^ T cell activation and proliferation (66). SnoRNA U50 is reduced in lymphocytes upon PHA stimulation, and its role in cell proliferation and rRNA transcription is mediated by the methylation of C2848 in 28S rRNA (67) lncSNHG2(GAS5) blocks the T-cell cycle, which is caused by snoRNA GAS5 mediated glucocorticoid receptor transcriptional activity *via* its decoy RNA “ glucocorticoid response element “ and mTOR activity (68). SNORA12 expression is downregulated in T cells from systemic lupus erythematosus (SLE) patients and regulates the expression of CD69, HIST1H4K by promoting interferon production of T cells (69). Zhong et al. showed that SnoRNA63 was increased and derived into piRNA(piR30840) to inhibit Th2 T cell development by downregulating IL-4 expression *via* sequence complementarity binding to pre-mRNA intron and mRNA decay (70). Beside the function in the T cell itself, SNORD116 and SNHG10 are found to be secreted by T cells in the CD47^+^ extracellular vesicles and therefore participate in cell communications between T cells (71, 72).

On the other hand, several lncSNHGs are reported to be upregulated during T cell activation, such as lncSNHG7, lncSNHG2 function as ceRNA to regulate T cell proliferation and activation (73, 74). Since these lncSNHGs are reported to be specifically correlated with the expression of certain snoRNA(s) (see **Table 1**), it is reasonable to suspect that the corresponding snoRNA(s) may play pivotal roles in regulating T cell function. However, there is rare research focused on the expression and regulation of snoRNA in T cell function, which could be an important issue to be addressed in the future. In addition, Chow et al. report that snoRNA-derived RNAs are significantly correlated with CD8^+^ T cell infiltration and cytolytic T cell activity (75), implying another critical role of snoRNAs in regulating T cell function *via* its downstream products.

At present, the expression of snoRNA in T cells in different physiological or pathological states, as well as in different T cell subtypes, is still unclear, especially the expression of snoRNA in tumor-infiltrated T cells and in malfunctioning T cells and their mechanisms of action are yet to be understood.

### B cell

SnoRNA plays important roles in the functional regulation of B cells. Compared to Germinal center, naïve, marginal zone and memory B-cells, peripheral blood B cells express a distinct snoRNA pattern with reduced SNORD116-1, SNORD116-23, SNORD116-29, SNORD94, and SNORA36A expression (76), indicating these snoRNAs may play a key role in B cell maturation and function. SnoRNAs derived from the DLK-DIO3 locus(containing 41 snoRNAs) were reduced whereas the expression of SNURF/SNRPN snoRNAs remained high in different types of B-cell malignancies compared to healthy B-cells (77). SNORA25 was reported to target 18S rRNA subunit (18S-801 and 18S:U814) and mediate the pseudouridylation of these residues (78). B cells are capable of secreting extracellular RNA, which is closely related to snoRNA content. In addition, Box C/D snoRNAs, SNORD123 and SNORD1a, were reported to be upregulated in B cells of aged mice and the implicated pathways including EIF2, mTOR signaling, p53, Paxillin, and Tec kinase signaling pathways, and cell cycle checkpoint, revealing the importance of snoRNA in B cell functions (79). In a study on chronic B-cell lymphocytic leukemia (CLL), 20 signature snoRNAs including SNORA80, SNORD1A, SNORD35B, SNORD71, SNORD116-11 and SNORD116-25 were found to be dysregulated in response to proliferation in CLL (80), confirming that snoRNAs play a crucial role in the functional regulation of B cells, and more importantly, function as biomarkers to discriminate between normal B-cells and CLL cases. To date, the vast majority of research focused on the expression profile of snoRNAs in B cells in both physiological and pathological conditions, although the expression pattern and clinical relevance of snoRNA in B cells are reported, the detailed mechanism of snoRNA in regulating B cell function and its immune modulating function remain not fully understood.

### Dendritic cell

As a professional antigen-presenting cell, DC is playing a pivotal role in tumor immunity. Rahmatpanah et al. found an enhanced DC activation state in healthy-aged individuals compared to young individuals. In these DCs, SNORA and SNORD were substantially upregulated from senescence onwards (81). Driedonks et al. found that immune-activating or inhibiting stimuli applied to primary DCs increased the levels of SNORD65 and SNORD68 snoRNA within extracellular vesicles (EVs). These findings demonstrated that DC-derived snoRNAs contribute to the communication of genetic information through EVs (82). In a melanoma model, snoRNA derived nuclear RNA3 (sdnRNA3) was found to be induced in DCs and may function to regulate iNOS expression *via* histone modification H3K27me3 at Nos2 gene promoter (83). At present, it is not clear whether environmental factors imposed on cells cause specific changes in a wide range of EV-associated snoRNAs. SnoRNA’s functional properties for EV in cellular communication and as a potential EV-RNA-based indicator of the immune status of EV-producing cells remain to be further investigated.

### Macrophage

SnoRNAs play important roles in macrophage polarization, activation, and intercellular communication. Ma et al. applied high-throughput sequencing and revealed that 121 snoRNAs were differentially expressed during M1 macrophage polarization and whereas 16 snoRNAs were significantly changed during M2 macrophage polarization. Particularly, snoRNA ENSMUST00000158683.2 was shown to inhibit the expression of TNF-α in macrophages. These findings suggest that snoRNA may be involved in the regulation of macrophage polarization and cytokine production (84). SnoRNAs affect tumorigenesis by regulating macrophage function through epigenetic modification of genes. Shi et al. identified a new sdnRNA, sdnRNA3, derived from snoRNA in M1 and M2 tumor-associated macrophages(TAMs). By recruiting the repressive chromatin-remodeling regulator Mi-2 and the repressive histone modification H3K27me3 at *Nos2* gene promoter, sdnRNA3 represses the transcription and expression of iNOS by repressing chromatin accessibility at the promoter of iNOS gene (83).Since TAMs are well-established to suppress anti-tumor immunity, these findings suggest nicely that snoRNA and sdnRNA3 could play pivotal roles in the formation of an immunosuppressive microenvironment. Although Chen et al. reported that lncSNHG2(GAS5) regulates apoptosis of macrophages after oxLDL treatment (85), it is unclear whether the corresponding snoRNAs have the same function in macrophages.

SnoRNA was also found to regulate the function of macrophages *via* vesicle-mediated intercellular communication. Rimer et al. reported that Rpl13a snoRNAs U32a (SNORD32a), U33 (SNORD33), U34 (SNORD34), and U35a (SNORD35a) were secreted by cultured mice and human macrophages upon activation. These snoRNAs were co-segregated with EVs and were taken up by recipient cells and direct new 2’-O-methylation on the 18S and 28S rRNAs in the recipient cell (86). These findings support a previously unappreciated link between inflammation and snoRNA secretion and reveal a potential role for secreted snoRNAs in intercellular communication. In addition, H/ACA small nucleolar ribonucleic acid protein particles (snRNPs) were found to be markedly expressed and regulate the growth arrest and differentiation of U937, a macrophage cell line, *via* down-regulation of NHP2 (87), suggesting a key role of snoRNA in the cell fate determination of macrophage.

Beside the immune cells, snoRNAs also act as a key player in cancer immunity *via* their regulation of immune checkpoint molecules. Programmed death (PD)-1 and Cytotoxic T-lymphocyte antigen 4 (CTLA-4) are immune checkpoint molecules that negatively regulate T-cell immune function (88). Monoclonal antibodies targeting these molecules have been approved by the US FDA to treat many types of cancer, therefore, it is of particular importance to understand the roles and mechanisms of snoRNAs in regulating PD-1/PD-L1 and CTLA-4 function. Xie et al. reported that different snoRNA signatures were highly associated with CTLA-4 and PD-1 expression in the high-risk group. In addition, patients with high snoRNA expression also display higher sensitivity to CTLA-4 and PD-1 inhibitors (89), suggesting that snoRNA could be a predictive molecule that assists in the treatment of patients with hepatocellular carcinoma.

In addition, snoRNAs are reported to be promising therapeutic and diagnostic targets for cancer. Zhu et al. identified nine snoRNAs signature (SNORA11B, SNORA36C, SNORA58, SNORA70J, SNORA75B, SNORD105B, SNORD126, SNORD3C and SNORD89) in ovarian cancer and reported these snoRNAs may serve as prognostic and therapeutic targets (90). Another snoRNA signature containing six snoRNAs(SNORA2, SNORA59B, SNORA70B, SNORD12B, SNORD93 and SNORD116-2) was reported to serve as a novel non-invasive biomarker for diagnosis and prognosis prediction of renal clear cell carcinoma (91). Interestingly, Cai et al. found 7 snoRNAs (SNORD59A, SNORD63B, SNORD100, SNORD99, SNORD63, SNORD12C, SNORD19) were reduced in tumors and were significantly increased in immune cells, these snoRNAs were identified as tumor immune infiltration-associated snoRNAs and can predict prognosis and immune landscape in patients with colon cancer (18). In addition to the signature of snoRNAs, single plasma snoRNAs such as SNORA71A, SNORD33 are also reported to serve as prognostic and diagnostic biomarkers for cancer (92, 93). Extracellular vesicles containing SNORA71E and SNORD115-6 can represent biomarkers for the prediction of the response of breast cancer patients to neoadjuvant chemotherapy (94). All mentioned studies have proposed snoRNA(s) as valuable predictive or diagnostic biomarkers, whether there are other snoRNAs in the liquid biopsy can serve as biomarkers for cancer diagnosis and prediction of anti-cancer immunity remains further investigation.

## LncSNHG-mediated anti-tumor immune responses

**Figure 4:**
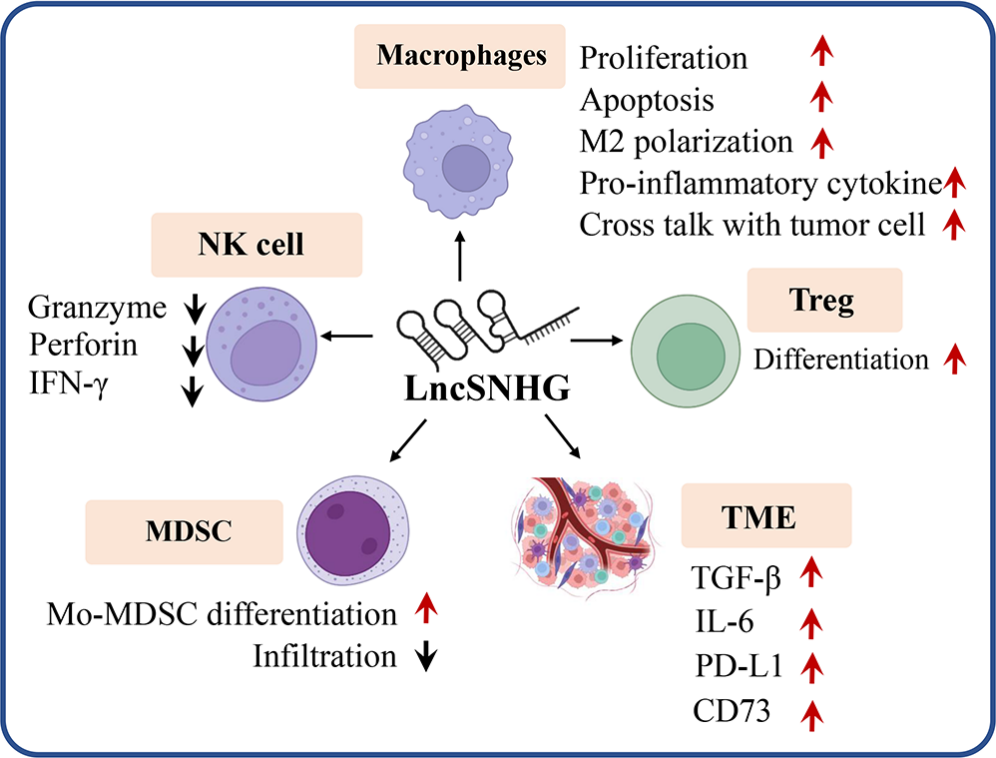
LncRNA are able to regulate a variety of immune cells (e.g. Treg, macrophages, MDSC, NK cells), immune checkpoints (e.g. PD-L1, CD73) and cytokines (e.g. TGF, IL-6) to influence tumor immunity. Red arrow, upregulation, black arrow, down regulation.

LncSNHG regulates immune cell function to participate in anti-tumor immunity. As shown in **Figure 4**, lncSNHG plays a pivotal role in anti-tumor immunity *via* its regulation in immune cells, such as regulatory T cells, macrophages, natural killer(NK) cells, and bone marrow-derived suppressor cells. LncSNHG regulates the direction of immune cell differentiation, the ability to secrete cytokines, and the proliferation, apoptosis, infiltration and interaction with tumor cells in the tumor microenvironment. In addition, lncSNHG can regulate the expression of immune checkpoints such as PD-L1, CD73 and cytokines such as TGF-β and IL-6 to participate in anti-tumor immunity. In the following section, we discuss the advance of lncSNHG in different immune cells.

### T cells

Regulatory T (Treg) cells are CD4^+^CD25^+^ T cells and specifically express Foxp3 protein (95). Studies have shown that lncSNHGs can promote the differentiation of regulatory T cells. Pei et al. found that lncSNHG1 was induced in the CD4^+^ tumor infiltrating lymphocyte(TIL), thereby sponging miR-448 to increase the expression of indoleamine 2,3-dioxygenase (IDO) and Foxp3 to regulate the differentiation of Treg cells and affect the immune escape of breast cancer (96). LncSNHG16 is reported to be secreted into tumor-derived exosomes and be uptaken by γδ1 T cells, which derepress the targeted SMAD5 by sponging miR-16-5p and its downstream CD73 expression to regulate γδ1 T cell proliferates into suppressive Treg population (97). To date, research on the expression and regulatory function of lncSNHGs in T cells is limited, it remains an open question that what is the expression profile of lncSNHGs in Tregs and other T cells, and what effect and mechanism do these lncSNHGs have to regulate the anti-tumor immunity of these T cells.

### Macrophage

Tumor-associated macrophages (TAMs) are one of the major infiltrating cells in the TME, where M1-type TAMs have tumor-killing effects while M2-type macrophages promote tumor progression (98). LncSNHG1 was reported to elevate the phosphorylation of STAT6 and therefore results in IL-4/IL-13 expression to promote M2 macrophage polarization, therefore, promote tumor development (99). Hu et al. report that lncSNHG1 can physically bind to high-mobility group box 1 (HMGB1), thus driving the inflammatory cytokines expression and macrophage activation (100). Qian et al. found that lncSNHG12 recruited NF-κB1 and promoted IL-6R transcription, and upregulation of SNHG12 promoted crosstalk between tumor cells and macrophages, eventually promoting the immune escape of breast cancer (101). LncSNHG16 was also shown to sponge miR-17-5p and elevate NF-κB signaling in promoting proliferation and inflammatory response in macrophages (102). LncSNHG15 can exert its function to prevent proinflammatory cytokine production, this is mediated by its direct interference with K63-linked ubiquitination of TNF-receptor-associated factor 2 (TRAF2) and inhibition of MAPK and NF-κB signaling pathways (103). LncSNHG2(GAS5) was shown to regulate macrophage apoptosis *via* activation of P53, Caspase 3, Caspase 7, and Caspase 9, however, the underlying mechanism remains unclear (85). In summary, lncSNHGs seem to affect almost all bioprocesses of macrophage, including proliferation, differentiation, apoptosis, pro-inflammatory response, and interaction with tumor cells.

There are several studies report that lncSNHGs are also involved in the cytokine release of macrophages. Zhang et al. showed that lncSNHG14 can promote misshapen-like kinase 1 (MINK1) expression through a ceRNA mechanism, ultimately increasing pro-inflammatory cytokine expression in rheumatoid arthritis (104).In LPS induced acute lung injury, lncSNHG14 was reported to be induced and mediate Wnt1 inducible signaling pathway protein 1 (WISP1) expression by sponging miR-34c-3p, thus promoting proinflammatory proteins IL-18, IL-1β, TNF-α and IL-6 (105).

Exosomal lncSNHG16 has been shown to directly target miR-140-5p and regulate the expression of TNF-α, IL-6, and IL-1β in macrophage, silencing lncSNHG16 inhibits macrophage proliferation and inflammation in combined Mycobacterium avium infections (106). Although this study proposes lncSNHG16 as a diagnostic biomarker for Mycobacterium tuberculosis-infection, the underlying mechanism needs to be investigated. To date, the existing research of lncSNHGs in macrophages has focused on the mechanism of ceRNA and protein binding and modification, whether other mechanisms like DNA methylation, mRNA splicing, etc., of a specific lncSNHG are involved in the functional regulation of macrophage remain further investigation.

### Bone marrow-derived suppressor cells

MDSC are mainly derived from bone marrow progenitor cells, and lncSNHG was found to play an important role in the regulation of MDSC function. It was shown that lncSNHG6 may be involved in regulating the differentiation of MDSCs by regulating the stability of EZH2 through the protein ubiquitination degradation pathway, but this study also showed that lncSNHG16 did not affect the immunosuppressive function of MDSCs (107). Also, lncSNHG was also found to be a key player in the infiltration of MDSCs. The SNHG6-miR-30e-5p-CYSLTR1 network was identified to be associated with prognosis in lung adenocarcinoma, where SNHG6 expression levels were negatively correlated with levels of neutrophils, macrophages, and DCs infiltration (108).

### Killer cells

LncSNHGs can promote apoptosis and suppress the function of tumor-killer cells such as CD4^+^ T lymphocytes, CD8^+^ T lymphocytes, and NK cells in tumor immunity. The apoptosis and infiltration of T lymphocytes caused by up-regulation of lncSNHGs expression were summarized in **Table 2**. Tumor cells promote PD-1/PD-L1 immune checkpoint triggering through upregulation of lncSNHGs, which can lead to apoptosis of CD4^+^ T cells (110), CD8^+^ T cells (111), and reduced infiltration (113). Particularly, lncSNHG12 is reported to reduce peripheral blood mononuclear cell proliferation and the ratio of CD8^+^ T cells by human antigen R (HuR)/ubiquitin-specific protease 8 (USP8) axis (114). LncSNHGs can also function as communication mediators between tumor cells and immune killer cells. The communication is mainly mediated by the extracellular vesical that cargo lncSNHGs. Huang et al. showed that lncSNHG10 secreted by tumor cells can upregulate INHBC expression in NK cells, thus suppressing the secretion of granzyme, perforin, and IFN-γ from NK cells by TGF-βactivation, and consequently diminishing the killing effect of NK cell (115). LncSNHG7 is embedded in mesenchymal stem cells (MSCs) derived exosome and affect the recipient cells *via* miR-34a-5p/XBP1 axis (116). Cancer stem cells also secret lncSNHG16 containing exosomes to influence other cells *via* lncSNHG16-TLR7 binding and MyD88/NF-κB/c-Myc activation. Since the exosomes derived from MSC and other cells are easily uptaken by NK cells and CD8^+^ T cells (117), it is reasonable to suspect that the exosomal lncSNHGs can influence the killer cell function *via* exosome-mediated intercellular communication.

**Table 2:** Effect of different lncSNHGs on PD-L1.

lncSNHGs	Types of cancer	Expression in Cancer	Regulatory site	PD-L1 expression	Impact	Ref.
**LncSNHG15**	Gastric cancer	up	miR-141	up		(109)
**LncSNHG4**	Colorectal cancer	up	miR-144-3p	up	CD4+T cell apoptosis	(110)
**LncSNHG14**	Diffuse large cell lymphoma	up	miR-5590-3p/ZEB1	up	CD8 + T cell apoptosis	(111)
**LncSNHG14**	Diffuse large cell lymphoma	up	miR-152-3p	up	Promoting TCL apoptosis	(112)
**LncSNHG1**	Renal cell cancer	up	miR-129-3p/STAT3	up	Inhibition of CD8+ T cell infiltration	(113)
**LncSNHG12**	Breast cancer	up	NF-κB1/IL-6R	up	Inhibition of T-cell infiltration	(101)
**LncSNHG12**	Non-small cell lung cancer	up	HuR/PD-L1/USP8	up	Inhibits PBMC, and CD8 T cells reduce TNF-α and IFN-γ levels and increase IL-10 and TGF-β levels	(114)

Tumor-infiltrating immune cells can regulate tumor progression and are important for the evaluation of clinical prognosis and immunotherapy (118). LncSNHGs are associated with immune infiltration in a variety of cancers and may be involved in cancer progression by regulating the function of immune cells. Chen et al. found that lncSNHG10 was increased and moderately associated with the infiltration of neutrophils, γδT cells, and macrophages in PC. They speculated that lncSNHG10 may inhibit the function of neutrophils and macrophages and promote the function of plasmacytoid dendritic cells (pDC) and NK cells, thereby promoting cancer progression (119). Li et al. also found that lncSNHG9 expression was negatively correlated with the level of infiltration of T central memory (Tcm) cells, and T helper cells in prostate cancer, and positively correlated with the level of infiltration of pDC and NK CD56 bright cells (120). In addition, Zhou et al. constructed six immune-related markers, including lncSNHG3, for predicting prognosis and immune infiltration in patients with hepatocellular carcinoma. They found that lncSNHG3 positively correlated with Th2 and Follicular T helper(Tfh) cells and negatively correlated with CD8^+^ T cells, Treg, and Th17 cells, and could regulate tumor progression (121).

Beside direct regulating the function of killer cells, lncSNHGs also function to regulate the expression and function of immune checkpoint molecules to contribute in immune-escape. A number of lncSNHGs have been reported to regulate the expression of PD-L1 *via* different mechanisms and pathways, which is summarized in **Table 2**. It seems that the most investigated mechanism of lncSNHGs on the immune checkpoint is *via* not only the ceRNA mechanism but also *via* interacting with transcription factors, participating in protein ubiquitination, and other molecular mechanisms. Zhou et al. reported that lncSNHG4 is upregulated in colorectal cancer and directly targets miR-144-3p to upregulate PD-L1 and ultimately increase apoptosis of CD4^+^ T cells (110). Tian et al. showed that tumor cells upregulate LncSNHG1 to promote STAT3-mediated PD-L1 expression and thus promote immune escape in renal cell carcinoma (113). As well, LncSNHG14 was reported to promote the activation of ZEB1, thus trans-activating SNHG14 and promoting the transcription of PD-L1 (111). LncSNHGs can also stabilize PD-L1 expression by participating in protein ubiquitination. lncSNHG12 binds to HuR and enhances the expression of PD-L1 with USP8, which improves PD-L1 stability by preventing ubiquitin-dependent degradation (114). CD73, an ectonucleotidase, is believed to be another key immune checkpoint molecule. LncSNHG was also shown to regulate the expression of CD73 in the TME, as Ni et al. showed that lncSNHG16 was packed into exosomes and acted on tumor-infiltrating lymphocytes (TILs) to upregulate the expression of CD73 molecules (97).

Although many studies are focusing on tumor or TME-derived lncSNHG on killer cell function, the expression and function of lncSNHG in the killer cell itself are still unclear. Whether lncSNHG regulates the function of T lymphocytes and NK cells, and the underlying mechanisms remain to be further investigated.

Beside the immune cells, lncSNHGs are also involved in the regulation of anti-tumor immune responses by regulating cytokine expression *via* different mechanisms and pathways. TGF-β can inhibit the anti-tumor effects of various immune cells in the TME (122). Recent studies have demonstrated that lncSNHG10 activates the TGF-β signaling pathway, leading to reduced secretion of granzyme, perforin, and IFN-γ from NK cells and diminished killing effect, resulting in immune escape from colorectal cancer (115). LncSNHGs have been shown to promote the EMT process by TGF-β (123). lncSNHG1, lncSNHG3, and lncSNHG6 have also been found to promote tumor proliferation migration and EMT processes *via* TGF-β or IL-6 signal pathway (124–126). The regulation of other tumor-associated cytokines by lncSNHGs and the impact of this regulation on tumor cells or immune cell production remains to be investigated.

The tight correlation of lncSNHGs with cancer also provides promising medical opportunities by targeting certain lncSNHGs. A systematic analysis based on 33 cancers revealed that lncSNHG3 and lncSNHG12 are closely associated with the prognosis of patients with multiple tumors (127). Patients with high lncSNHG7 and lncSNHG12 levels were reported to be correlated with longer and shorter overall survival (OS) and disease-free survival, respectively (15). LncSNGH4, lncSNHG5, lncSNHG7, and lncSNHG12 were also reported to be potential therapeutic targets and biomarkers for human cancers (128–131). LncSNHG9 is strongly correlated with poor immune infiltrations and progression-free survival, suggesting its role as a promising prognostic biomarker in prostate cancer (120). In addition, silencing the expression of lncSNHG15 could inhibit the proliferation and the migration of breast cancer, thus negating cisplatin resistance and providing novel therapeutic strategies for breast cancer (132). Silencing lncSNHG12 can restrict tumor growth and upregulate the ratio of CD8^+^ T cells, suggesting lncSNHG12 could be a potential therapeutic target (114). The circulating lncRNAs could be a source of cancer liquid biopsy biomarkers, lncSNHG2(GAS)in the plasma is suggested to be a diagnostic biomarker for multiple myeloma (133). LncSNHG1 and lncSNHG18 in the plasma are reported to serve as diagnostic biomarkers for hepatocellular carcinoma (134, 135). Meanwhile, lncSNHG15 is suggested as a highly specific and sensitive biomarker for diagnosis of acute myeloid leukemia (136). All these researches provide us a therapeutic and diagnostic view of lncSNHGs for cancer treatment and diagnosis, however, the clinical application of these molecules still needs further verification.

## Conclusions

snoRNA and lncSNHG are the transcripts derived from the SNHGs with or without protein-coding potential, and these two types of ncRNA are diversely expressed in a variety of tumor and immune cells. Since cancer immunotherapy has emerged based on the critical role of different types of immune cells, it is of particular importance to understander how snoRNA and lncSNHGs are changed and the detailed mechanisms underlying snoRNA and lncSNHGs-mediated immune cell function in anti-tumor immunity. In this review, we have summarized the expression of snoRNAs and lncSNHGs in distinct immune cell types under certain clinical circumstances. We reviewed the canonical and noncanonical mechanism of snoRNA to regulate anti-tumor immunity *via* influencing the function of immune cell function and infiltration, cytokines production, and immune checkpoints expression. Meantime, we have summarized the roles and mechanisms of lncSNHGs in regulating the immune cell function to suggest an ignored role of lncSNHGs in anti-tumor immunity. As a number of studies have focused on the clinical relevance and their potential application in cancer, the literature review strongly supports that snoRNA and lncSNHGs may be promising biomarkers and treatment targets for cancer immunotherapy. However, there are several open questions remain to be addressed:

- What are the precise expression pattern and spatiotemporal relationships of snoRNA and lncSNHGs with immune cell status, and what is the role of a specific snoRNA and lncSNHG play in the dysregulated immune cells that exert anti-tumor immunity?
- What is the role of snoRNA and lncSNHG in adoptively transferred immune cells, as well as in the immune cells after immune checkpoint inhibitor treatment, and how these molecules can be utilized to enhance the anti-tumor function and reduce adverse events?
- The immune system itself and patients display diverse individual variations, the expression pattern of snoRNA and lncSNHGs may vary drastically from patient to patient, or even from different disease stages of the same patient. This is the biggest challenge of clinical application of targeting snoRNA and lncSNHGs.

By answering these questions, we can therefore better optimize the strategy of utilizing snoRNA and lncSNHGs as treatment or diagnostic targets for cancer patients. In addition, profiling the expression pattern of these ncRNAs in the liquid biopsy and the tissues would help us to better personalize the diagnostic and treatment strategies.

## Author contributions

HX: contributed to the central idea, wrote and revised the paper, and drew the figures. XF: contributed to the central idea, wrote and revised the paper, and drew the figures. ML: contributed to refining the ideas and finalizing this paper and drew the figures. HG: contributed to refining the ideas and finalizing this paper and proofread the paper. XZ: contributed to the central idea, touched up and revised the paper, and proofread the paper. All authors contributed to the article and approved the submitted version.
